# Editorial: Women in ethnopharmacology: 2023

**DOI:** 10.3389/fphar.2024.1541890

**Published:** 2025-01-06

**Authors:** Marilena Gilca

**Affiliations:** Department of Functional Sciences I/Biochemistry, Faculty of Medicine, Carol Davila University of Medicine and Pharmacy, Bucharest, Romania

**Keywords:** women, ethnopharmacology, endometriosis, TCM, medicinal plants

Despite the fact that the gender landscape in education and the workforce in terms of women’s representation and recognition has improved lately, differences persist in various science-related fields (Charlesworth and Banaji, 2019), with women more likely to experience non-standard professional trajectories and negative health effects (Cabib et al., 2024).

Recognizing the importance of cultivating gender equality for sustainable development, as highlighted by UNESCO, we initiated a Research Topic to provide a collaborative platform to promote the work of women scientists in all fields of Ethnopharmacology. In response to our call for submissions, 21 articles were submitted, of which only 11 met publication standards.

In the following section of this editorial, some important achievements in the field will be highlighted, and also some Research Topic for future research will be identified.

One of the important health Research Topic affecting 10% of the women of reproductive age is endometriosis (Shafrir et al., 2018). In addition to its negative impact on the quality of life, it also represents an economic burden (Swift et al., 2024). A systematic review and meta-analysis performed by Ding et al. concluded that traditional Chinese Herbal Medicines (CHMs) may be a viable postoperative long-term therapeutic strategy for ovarian endometriotic cysts, and may improve pain, pregnancy rate, perimenopausal symptoms, and serum levels of Cancer antigen 125 while reducing recurrence and adverse effects of conventional Western medicine.

Despite the increasing global popularity of CHMs, there are several concerns due to their potential health risks, toxicities, risks of poor quality and adulteration. Difficulties in quality control of CHMs are also due to the complex and diverse processing of Chinese herbal ingredients. Mück et al. addressed these Research Topic and found an undesirably high level of variability in two traditional Chinese herbal formulas used in women’s healthcare for the treatment of endometriosis, known as *Gui Zhi Fu Ling Wan* and *Ge Xia Zhu Yu Tang,* which are already available on the European market. Mück et al. showed in another study that conventional DNA barcoding is an insufficient tool for authentication of these samples and proposed a new tiered identification strategy based on HPTLC and DNA metabarcoding, which gives a progressive qualitative and quantitative insight on species diversity, and biological contaminants.

Inflammatory pathways are key components of many pathologies of the female reproductive tract, including endometriosis, polycystic ovary syndrome, etc., and their hyperactivity or dysregulation contributes to the onset of these disorders (Jabbour et al., 2009). Three articles in our Research Topic identified some potential natural anti-inflammatory agents. Nguyen et al. demonstrated the potential of *Acer tegmentosum* Maxim extract and its fermented product to prevent endothelial inflammation and vascular dysfunction of the retina through the MAPK/NF-κB/SIRT1 signaling pathways. Cebollada et al. evaluated the 5-lipoxygenase inhibitory activity of several essential oils and showed that those derived from clove (*Syzygium aromaticum* (L.) Merr. and L.M.Perry) and wintergreen (*Gaultheria fragrantissima* Wall.) were the most active. Another study conducted by Fabian et al. demonstrated the 15-lipoxygenase and α-glucosidase inhibitory activity of several bioactive fractions obtained from the stem of *Coriaria intermedia* Matsum. and the bark of *Dracontomelon dao* (Blanco) Merr. and Rolfe. *C.,* which were generated, and dereplicated through UHPLC-MS/MS. The authors identified corilagin as a potential anti-inflammatory and anti-diabetic agent that should be prioritized in further studies.

Previous studies have shown that women seem to have a special relationship with traditional medicine and are among its most active consumers. Luo et al. re-confirmed this fact in a cross-cultural study exploring the traditional animal- and mineral-based medicines that are used in the Gansu-Ningxia-Inner Mongolia junction zone, a region of ethnic and cultural diversity. They found that female informants demonstrated greater knowledge of the medicines, probably due to their prevalence among the local herbal practitioners.

Taking into account that women play an essential role in the primary healthcare of children, and, at the same time, that ethnopediatric practices are poorly documented, Petran et al. focused on the study of medicinal plants currently employed in the treatment of childhood illnesses in the southern region of Romania. Higher education correlated not only with the number of plants employed and the variety of ailments treated but, surprisingly, also with the preference for harvesting rather than purchasing plants. The authors also raised concerns regarding the necessity to protect this ethnomedical heritage, since there is a significant reduction in the used taxa when compared to the past.

Although not statistically significant, women with a lower socioeconomic status had a higher cardiovascular risk than men (Ololade et al., 2024). Also, women with polycystic ovary syndrome, a frequent endocrinopathy, have a higher chance of developing a cardiovascular disease (Dutta and Maddukuri, 2024), while the leading cause of mortality in women with breast cancer is represented by the same type of cardiovascular pathology (Jiao et al., 2024).

Given the importance of cardiovascular health to women’s wellbeing, some space was given to this area in the present Research Topic. Wang et al. provided a systematic review of the efficacy and safety of anisidine hydrobromide injection for acute ischemic stroke. Anisodine is a tropane alkaloid extracted from the root of *Anisodus tanguticus (Maxim.) Pascher,* family Solanaceae. It can improve significantly cerebral collateral circulation and increase blood flow perfusion in ischemic areas, and has been used for the treatment of ischemic stroke in clinical settings in China for more than a decade.

Blackthorn flower (*Prunus spinosa* L.) is a traditional remedy recommended for treating cardiovascular disease. Flavonol and A-type procyanidin-rich extracts of this medicinal plant exhibited anticoagulant activity through direct thrombin inhibition, without affecting platelet aggregation *in vitro,* according to Marchelak et al.

The studies presented here highlight the diversity of research performed across the breadth of Ethnopharmacology and present some advances in knowledge with applications to compelling women-related health Research Topic, such as endometriosis, and cardiovascular and inflammatory diseases, while identifying problems that require attention, such as quality control of CHMs and preservation of biocultural heritage.

The editors expect that the articles published in this Research Topic will have a significant impact on the readers and believe that more women scientists will be thus encouraged to be involved in ethnopharmacological research, contributing to the development of new effective therapeutic approaches inspired by traditional medicine.
